# Suppression of Aurora-A-FLJ10540 signaling axis prohibits the malignant state of head and neck cancer

**DOI:** 10.1186/s12943-015-0348-7

**Published:** 2015-04-12

**Authors:** Chang-Han Chen, Alice YW Chang, Shau-Hsuan Li, Hsin-Ting Tsai, Li-Yen Shiu, Li-Jen Su, Wen-Lung Wang, Tai-Jen Chiu, Sheng-Dean Luo, Tai-Lin Huang, Chih-Yen Chien

**Affiliations:** Center for Translational Research in Biomedical Sciences, Kaohsiung Chang Gung Memorial Hospital, Kaohsiung, Taiwan; Department of Otolaryngology, Kaohsiung Chang Gung Memorial Hospital, and Chang Gung University College of Medicine, Kaohsiung, Taiwan; Kaohsiung Chang Gung Head and Neck Oncology Group, Kaohsiung Chang Gung Memorial Hospital, Kaohsiung, Taiwan; Institute of Physiology, National Cheng Kung University, Tainan, Taiwan; Departments of Hematology-Oncology, Chang Gung University College of Medicine, Kaohsiung, Taiwan; Department of Medical Research, Cell Therapy and Research Center, E-Da Hospital, I-shou University, Kaohsiung, Taiwan; Graduate Institute of Systems Biology and Bioinformatics, National Central University, Jhongli, Taiwan; Department of Applied Chemistry and Graduate Institute of Biomedicine and Biomedical Technology, National Chi Nan University, Taoyuan, Taiwan; Department of Medical Research, Kaohsiung Chang Gung Memorial Hospital, Kaohsiung, Taiwan

**Keywords:** HNC, Aurora-A, FLJ10540, MMP-7 and MMP-10

## Abstract

**Background:**

Head and neck cancer (HNC) is a highly invasive cancer. Aurora-A has been reported for a number of malignancies. However, the identity of downstream effectors responsible for its aggressive phenotype in HNC remains underinvestigated.

**Methods:**

The mRNA and protein expression levels of Aurora-A and FLJ10540 were assessed in HNC specimens and cell lines using RT-qPCR, western blot, Oncomine, and microarray database analysis. The downstream molecular mechanisms of Aurora-A were confirmed by RT-qPCR, western blot, luciferase reporter, confocal microscopy analyses, immunoprecipitation, colony formation, cell viability, and xenograft model. Cellular functions in response to Aurora-A-modulated downstream targets such as FLJ10540 and MMPs were examined *in vitro* and *in vivo*, including cell growth, motility and chemosensitivity. Aurora-A/FLJ10540/MMPs expression was determined in cancer and adjacent normal tissues from HNC patients by immunohistochemistry approach.

**Results:**

In the current study, *Aurora-A* exhibited similar gene expression profiles with *FLJ10540* by using accessibly public microarray and Oncomine database analysis, raising the possibility that these molecules might coordinately participate in cancer progression and metastasis of HNC. These two molecules connection were also examined in cell lines and tissues of HNC. Aurora-A overexpression could not only bind to the promoter of FLJ10540 to induce FLJ10540 expression, but also increase both mRNA and protein levels of MMP-7 and MMP-10 in HNC cells. Conversely, depletion of Aurora-A expression by using siRNA or Aurora-A kinase inhibitor, MLN8237, suppressed FLJ10540, MMP-7 and MMP-10 mRNA and protein expressions *in vitro* and *in vivo*. In addition, the FLJ10540-PI3K complex was destroyed by inhibition the Aurora-A kinase activity. Forced overexpression of FLJ10540 in Aurora-A-depleted or in MLN8237-treated HNC cells attenuated the effect on cytotoxicity to cisplatin. Elevated Aurora-A expression in HNC cells led to the characteristics of more aggressive malignancy, including enhanced chemoresistance and increased the abilities of proliferation, migration and invasion, which was required for FLJ10540/MMP-7 or FLJ10540/MMP-10 expressions. Finally, immunohistochemical analysis of human HNC specimens showed a significant positively correlation among Aurora-A, FLJ10540, MMP-7 and MMP-10 expressions.

**Conclusion:**

Together, our findings define a novel mechanism by which Aurora-A promotes cell malignancy, with potential implications for understanding the clinical action of Aurora-A.

**Electronic supplementary material:**

The online version of this article (doi:10.1186/s12943-015-0348-7) contains supplementary material, which is available to authorized users.

## Introduction

Head and neck cancer (HNC) is the sixth most common cancer worldwide, the incidence of HNC is estimated at 560,000 new cases and 300,000 deaths annually [1]. Approximately 90% of HNC arises in the mucosa of the oral cavity, oropharynx, larynx and hypopharynx. According to a histopathological perspective, more than 90% of HNC are squamous cell carcinomas [2]. Both environmental factors and genetic inheritance can give rise to the development of HNC. Tobacco, alcohol consumption and areca nut are major risk factors for the development of this disease. Patients with early stage can be cured by therapy; however, two-third of HNC patients with advanced disease at time of diagnosis, due to HNC has a high potential for local recurrent, invasion and lymph node metastasis [3]. The most common treatment modalities for HNC include surgery, radiation, and chemotherapy, often in combination. Despite new treatment options for HNC patients, the 5-year mortality rate has not improved over the past 3 decades. Therefore, investigations specifically aimed at further understanding the molecular basis involving in HNC carcinogenesis can facilitate the integration of diagnosis and therapy for HNC in the future.

Aurora-A, also designated as STK15/STK6, is a serine/threonine kinase that plays a crucial role in mitosis and spindle assembly during various stage of mitosis. Aberrant Aurora-A amplification and/ or overexpression has been reported in human malignancies, such as colon, neuroblastoma, breast, oral, NPC, pancreas, ovary, lung, esophagus cancers [4-13]. Increased Aurora-A expression may cause genomic instability, tumorigenesis, metastasis and chemoresistance, correlating with its pro-survival function in cancer cells [14]. Accumulated reports indicate that evaluated Aurora-A expression is not only correlated with advanced stage of tumors, but also associated with a poorer outcome of patients. Recent studies have shown that oncogenic signaling pathways, such as GSK-3β, c-Myc, AKT, β-catenin, p53 and NF-κb involved in Aurora-A function in cancers [15]. Based on these results, Aurora-A plays a key converging point of a complex network of oncogenic signaling pathway. However, the role of Aurora-A in the signaling transduction pathway involved in tumorigenesis of HNC has not been fully clarified.

FLJ10540, a protein of 464 amino acids, has been mapped to the 10q23 chromosomal region. This protein contains three coiled-coil domains, a peroxisomal targeting signal 2, a nuclear export signal, and structure maintenance of chromosome (SMC) domain. Frequent overexpression of FLJ10540 has been reported in HCC, lung, OSCC, and NPC [16-20]. FLJ10540 is a mitotic phosphoprotein and has been reported to be an essential regulator of cytokinesis controlled by Cdk1 and ERK2 [21]. Deregulated expression of FLJ10540 may cause cytokinesis defects, genetic instability and oncogenic transformation [22]. Highly FLJ10540 expression promotes progrowth signaling pathways resulting in cancer cell proliferation, metastasis and poor patient prognosis in human cancers [16] [17,20]. Therefore, the oncogenic potential and essential roles in cell cycle of FLJ10540 make it an intriguing target for anticancer therapeutic intervention.

In the present study, we demonstrate for the first time that Aurora-A induced FLJ10540 expression not only influences FLJ10540/PI3K complex, but also regulates MMP-7 and MMP-10 activations, thereby leading to the proliferation, metastasis of HNC cells and their resistance to cisplatin treatment in HNC.

## Materials and methods

### Reagents

MLN8237 and GM6001 were purchased from Selleck Chemicals. Cisplatin was purchased from Sigma-Aldrich. All chemicals were dissolved in dimethyl sulfoxide (DMSO) for *in vitro* studies.

### Human HNC tissue samples and IHC

Commercially purchased tissue microarrays (TMAs) included 80 samples of 11 cases in early stage, 59 cases in advanced stage and 10 normal tissue (US Biomax, Inc., Rockville, MD, USA; catalog number HN802). This study was approved by the Medical Ethics and Human Clinical Trial Committee at Chang Gung Memorial Hospital. Tissues were fixed with 10% buffered formalin embedded in paraffin and decalcified in 10% EDTA solution. Representative blocks of the formalin-fixed, paraffin-embedded tissues were cut to 4 mm and deparaffinized with xylene and rehydrated in a series of ethanol washes (100, 90, 80, and 70%). Slides were washed with phosphate-buffered saline (PBS) and treated with 3% H_2_O_2_ for 30 minutes to block endogenous peroxidase activity. Next, the sections were microwaved in 10 mM citrate buffer, pH 6.0, to unmask the epitopes. After antigen retrieval, the sections were incubated with diluted anti-Aurora-A, anti-FLJ10540, anti-MMP-7 and anti-MMP-10 antibodies for 1 h followed by washing with PBS. Horseradish peroxidase/Fab polymer conjugate (PicTure™-Plus kit; Zymed, South San Francisco, CA, USA) was then applied to the sections for 30 min followed by washing with PBS. Finally, the sections were incubated with diaminobenzidine for 5 min to develop the signals. A negative control was run simultaneously by omitting the primary antibody. The reactivity level of the immunostained tissues was evaluated independently by two pathologists who were blind to the subjects’ clinical information. Between 15 and 20 high-power fields were viewed. Criteria were developed for quantitating the immunoreactivities of the Aurora-A, FLJ10540, MMP-7 and MMP-10 stainings in both the normal and tumor sections using a score range of 0 to +3, where 0 indicated no positive cell staining, +1 less than 10% positive cell staining, +2 10-30% positive cell staining, and +3 more than 30% positive cell staining. Similarly, the stain intensity was graded as +0, +1, +2, or +3 as previously described [23].

### Cell culture, transient transfection, the establishment of stable clones, and luciferase assay

FaDu and SAS cell lines were obtained from the American Type Culture Collection. All cell culture-related reagents were purchased from Gibco-BRL (Grand Island, NY, USA). FaDu and SCC4 cells were grown in DMEM containing 10% FBS and 100 U/ml penicillin and streptomycin (Gibco-BRL) Flag-vector (pcDNA3.1), Flag-Aurora-A and Flag-FLJ10540 were transiently transfected into cancer cells using Lipofectamine (Invitrogen) according to the manufacturer’s instructions. FaDu cells mixed-stably expressing Aurora-A or FLJ10540 were selected with 400 μg/ml G418 (Calbiochem Novabiochem, San Diego, CA, USA) for two weeks. The cell were then harvested and analyzed for exogenous Aurora-A and FLJ10540 expressions by Western blotting. 5′-upstream fragments of *FLJ10540* gene (−1 ~ −2000) was amplified from human genomic DNA and verified by sequencing. The PCR fragments were cloned into firefly luciferase reporter vector pGL3-Basic (Promega) NheI and HindIII sites which were designed into the forward and the reverse primers, respectively. For co-transfection experiments, FaDu cells were co-transfected with 100 ng firefly luciferase reporter plasmids (pGL3-Basic or pGL3-FLJ10540), and 10 ng of pRL-TK *Renilla* luciferase internal control plasmid. After 24 h, the luciferase activity was measured using Dual Glo™ Luciferase Assay System (Promega). Two double-stranded synthetic RNA oligomers (5′-GCAGAGAACUGCUACUUAUtt-3′ deduced from human Aurora-A; and 5′-GGACTTTTAGCAAAGATCTtt-3′ deduced from human FLJ10540; Ambion; Taipei, Taiwan) deduced from human *Aurora-A*, and one negative control siRNA (#4611G; Ambion) were used in the siRNA experiments.

### Immunoblot analysis

For tissue protein extraction, frozen samples were homogenized in RIPA lysis buffer (50 mM Tris–HCl, pH 7.5, 150 mM NaCl, 1% NP-40, 0.5% Na-deoxycholate, and 0.1% SDS). The protein concentration in each sample was estimated by Bio-Rad Protein Assay (Bio-Rad, Hercules, CA, USA). Immunoblotting was performed according to standard procedures. Antibodies used in this study include Aurora-A (monoclonal; Epitomics, Burlingame, CA, USA), MMP-7 (monoclonal; Millipore), MMP-10 (monoclonal; Millipore), FLJ10540 (generated by us) and β-actin (monoclonal; Santa Cruz Biotechnology, Santa Cruz, CA, USA). The first antibodies were detected by incubation with secondary antibodies conjugated to HRP (Bio/Can Scientific, Mississauga, ON, Canada) and developed using Western Lighting Reagent. The proteins were explored by X-ray films. The protein expression levels were quantified by ImageJ Software and represented as the densitometric ratio of the targeted protein to GAPDH or β-actin.

### Indirect immunofluorescence analysis and microscopy

The indirect immunofluorescence staining on the HNC cells treated with MLN8237 or Aurora-A/siFLJ10540 was performed with anti-FLJ10540, at RT for 2 h. The sections were then washed three times with PBST and incubated with DAPI and goat-anti-rabbit-FITC (Jackson, ImmunoResearch) at RT for 1 h. After washing with PBST, the sections were mounted with GEL/Mount (biomeda corp, Foster, CA). The fluorescence images on the slips were examined using a confocal microscope (Olympus FV10i).

### RNA extraction, semi-quantitative RT-PCR, and quantitative RT-PCR

Samples were frozen in liquid nitrogen and stored at −80°C prior to RNA extraction. The cells were homogenized using a Mixer Mill Homogenizer (Qiagen, Crawley, West Sussex, UK). Total RNA was prepared from the frozen tissue samples using an RNeasy Mini Kit (Qiagen) according to the manufacturer’s instructions. The RNA (2 μg) was then reverse transcribed into cDNA using SuperScript II Reverse Transcriptase (Invitrogen, Carlsbad, CA, USA). For Q-RT-PCR, *Aurora-A*, *FLJ10540* and *MMPs* Taq-Man probe (ABI) were used to perform the study. Data were represented as mean ± s.d. To analyze the distribution of control and experimental groups, we performed the Wilcoxon signed rank test between two groups for statistical analysis. A *P*-value of less than 0.05 was significant. *GAPDH* (ABI) was used as an internal control for comparison and normalization the data. Assays were performed in triplicate using Applied Biosystems Model 7700 instruments.

### Migration, and invasion assays

Migration and invasion assays were conducted with FaDu-vehicle, FaDu/Aurora-A/negative, FaDu/Aurora-A/siFLJ10540, FaDu/Aurora-A/DMSO, and FaDu/Aurora-A/GM6001 cells using 24-well Transwell chambers (8-μm pore size polycarbonate membrane; Costar, Corning, NY). For the migration (5 x 10^3^) and invasion (1 x 10^4^) assays, cells were suspended in 400 μl of DMEM containing 10% FBS, then seeded into the upper chamber; 600 μl of DMEM containing 10% FBS were added to the outside of the chamber. After being cultured at 37°C under 5% CO_2_/95% air for 24 h, the cells on the upper surface of the membrane were removed with a cotton-tipped applicator and the migratory cells on the lower membrane surface were fixed with methanol and stained with Giemsa (Sigma, USA). Cell migration was evaluated by counting the number of FaDu-vehicle, FaDu/Aurora-A/negative, FaDu/Aurora-A/siFLJ10540, FaDu/Aurora-A/DMSO, and FaDu/Aurora-A/GM6001 cells that had migrated by 200× phase-contrast microscopy on three independent membranes, then normalized against the vehicle cells to determine the relative ratio. For the invasion assays, 80 μg/ml of Matrigel (BD Biosciences) were added to the upper surface of the membrane and allowed to gel at 37°C overnight. A total cells (1×10^5^) in 400 μl of DMEM containing 10% FBS were seeded into the upper chamber, while 600 μl of DMEM containing 10% FBS were added to the outside of the chamber. The rest of the protocol was the same as that for the migration assays.

### Chromatin immunoprecipitation (ChIP)

ChIP assays were performed according to the protocol from Millipore (EZ–Magna ChIP G Chromatin Immunoprecipitation Kit, Millipore). FaDu cells transfected with vehicle control and HA fused Aurora-A according to the manufacturer’s instruction that was describe above. Chromatin was precipitated using anti-HA antibody and protein A agarose at 4°C overnight and immune complexes were collected by centrifugation. Normal human IgG was used as a control. Cross-links were then reversed at 65°C overnight. The purified DNA was amplified by PCR using FLJ10540 promoter primers pre-denaturation for 3 min at 94°C, denaturation at 94°C for 20 sec., annealing at 47°Cfor 30 sec., and extension at 72°C for 30 sec. for a total of 30 cycles).

### Measurement of MMP-7 and MMP-10 protein

The amount of MMP-7 and MMP-10 proteins in the conditioned media was determined using the human MMP-7 and MMP-10 Quantikine ELISA kits (R&D Systems, Minneapolis, MN, USA) according to the manufacturer’s instructions. The amounts of MMP-7 and MMP-10 were calculated from a standard curve.

### Cell viability assay and colony formation assay

Viability of sub-confluent cells was analyzed by 3-(4,5-dimethylthiazole-2-yl)-2,5-diphenyltetrazolium bromide (MTT) reduction assay. FaDu/vehicle + negative control, FaDu/vehicle + siFLJ10540, FaDu/Aurora-A + negative control, FaDu/Aurora-A + siFLJ10540, SAS/negative control, SAS/vehicle + negative control, SAS/FLJ10540 + negative control, SAS/siAurora-A, SAS/vehicle + siAurora-A, and SAS/FLJ10540 + siAurora-A cells were seeded at 5 × 10^3^ cells/well in 96-well plates. Next day, cells were treated with MLN8237 (0.025 nM) or cisplatin (15 μM) or in combination for 48 h. After that, MTT solution was added to each well. The plates were stored at 37°C for 4 hour, and then 100 μL DMSO buffer was added and incubated in the dark for 10 min. Absorbance was measured on a microplate reader at 540 nm. The OD values were normalized with the value of control group. For colony formation assay, cells were seeded in 60-mm dishes at a density of 5 × 10^3^ cells. Next day, cells were treated with MLN8237 (0.025 nM) and cisplatin (15 μM) or GM6001 (3 μM) for 48 h. After washing with PBS, the cells were incubated in drug-free complete medium for 15 days. Subsequently, cell colonies were counted after staining with 0.01% crystal violet.

### Animal experiments and immunohistochemistry

Parental SAS, SAS/negative control and SAS/siFLJ10540 cells were harvested, washed in PBS, and suspended in a mixture of PBS and Matrigel (BD Biosciences, San Jose, CA, USA). 1 × 10^6^ cells were injected into the flanks of female nude mice. All animal experiments were carried out in accordance with protocols approved by the Animal Use and Management Committee of Kaohsiung Chang-Gung Memorial Hospital. After tumor growth reached 100 mm^3^, mice were assigned to receive MLN8237 or DMSO by oral gavage for 14 days. Mice were monitored daily and tumor volumes and body weights were measured twice weekly. At the completion of the study, tumors were excised, formalin-fixed and paraffin-embedded for immunohistochemical analysis.

### Statistical analysis

All *in vitro* experiments were performed in triplicates. ANOVA analysis was used to evaluate statistical difference between groups. The correlation between two parameters was determined by the Spearman correlation. The *P* value of 0.05 or less was considered statistically significant.

## Results

### FLJ10540 and Aurora-A are abnormally co-expressed in HNC tumor tissues and cell lines

**Figure 1:**
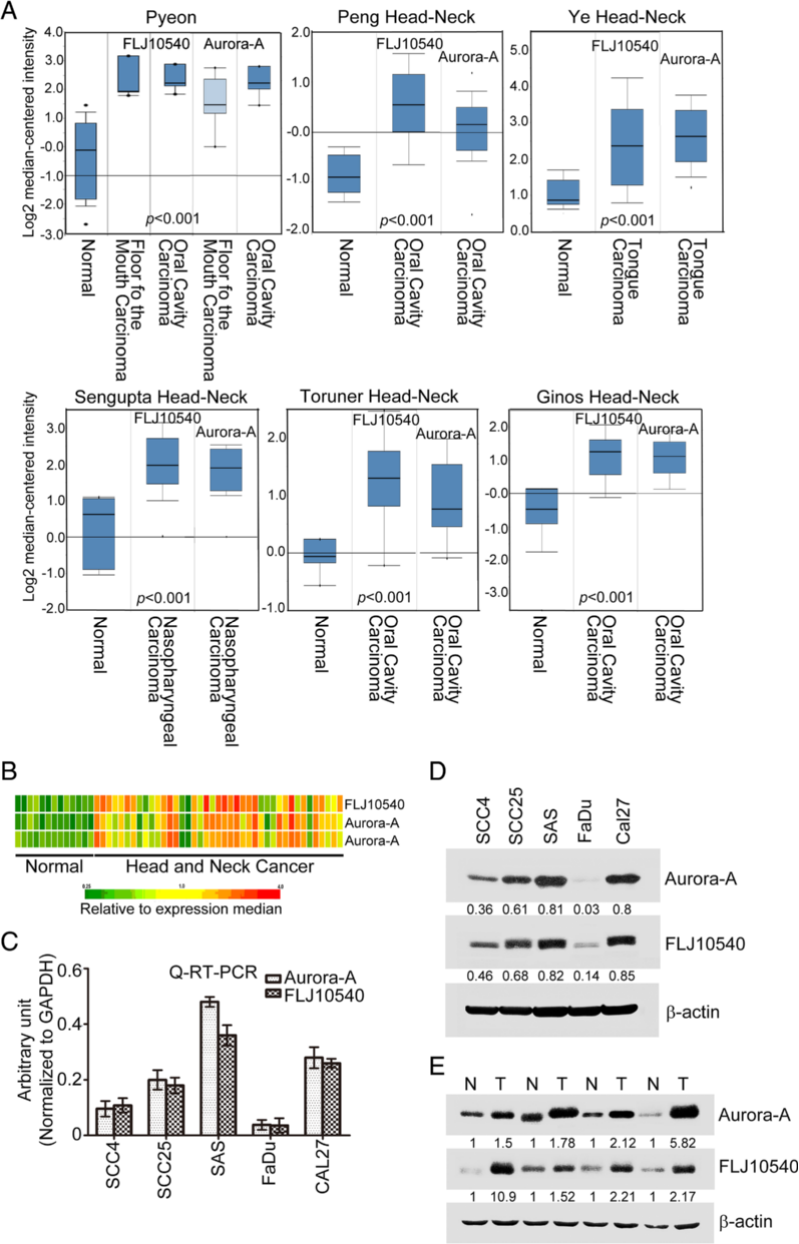
Clinical and cellular significances of FLJ10540 and Aurora-A in HNC. (**A**) Oncomine data mining analysis of *Aurora-A* and *FLJ10540* mRNA levels in six different datasets between normal tissues versus head and neck carcinomas. (**B**) The correlation between the relative expression levels of FLJ10540 and Aurora-A in microarray database of HNC. (**C** and **D**) The mRNA and protein expression levels of FLJ10540 and Aurora-A in HNC cells were analyzed by Q-RT-PCR and Western blotting. GAPDH and β-actin were used as internal control. (**E**) Western blotting analysis of FLJ10540 and Aurora-A expressions were determined in paired HNC patients. Total proteins were extracted from adjacent non-cancerous and tumor tissues and probed with polyclonal antibodies against Aurora-A and FLJ10540. β-actin was used as a control. Images were quantified with ImageJ software.

Previous reports have indicated that either Aurora-A or FLJ10540 involves in multiple signaling pathways to promote cancer progression [12,13,15,17,24,25]. Intersection of these pathways elicited by either Aurora-A or FLJ10540, we found that these two molecules have shared common signal pathways for subsistence of tumor cells. First, to determine the clinical significances of both Aurora-A and FLJ10540 in patients with HNC, we performed data mining and analyzed both Aurora-A and FLJ10540 expressions from the publicly available Oncomine database. The finding for Aurora-A and FLJ10540 gene expressions based on 13 databases exhibited six with a significant p-value (p < 0.001) and gene ranks in the top 10% among all differentially expressed genes. In these six databases, Aurora-A and FLJ10540 were up-regulated in tumor tissues of HNC compared with normal tissues (Figure 1A). Next, from cDNA microarray analysis using samples of HNC specimens, we also observed that *FLJ10540* was highly positively correlated with the mRNA expression level of *Aurora-A* in specimens of HNC patients (r = 0.72, p < 0.001) (Figure 1B). These results raise the possibility that Aurora-A and FLJ10540 have functionally linked in human cancers. To this end, we first examined the expression levels of Aurora-A and FLJ10540 in HNC cell lines. It was indicated that the mRNA and protein expression levels of Aurora-A were paralleled the levels of increased FLJ10540 mRNA and protein as determined by Q-RT-PCR and Western blotting (Figure 1C and D). In addition, we further investigated Aurora-A and FLJ10540 protein expression profiles in paired specimens of HNC. The result demonstrates that both Aurora-A and FLJ10540 expressions are paralleled elevated in HNC specimens (Figure 1E), suggesting a potential functional association between Aurora-A and FLJ10540 in HNC.

### FLJ10540 expression is modulated by Aurora-A in HNC cells

**Figure 2:**
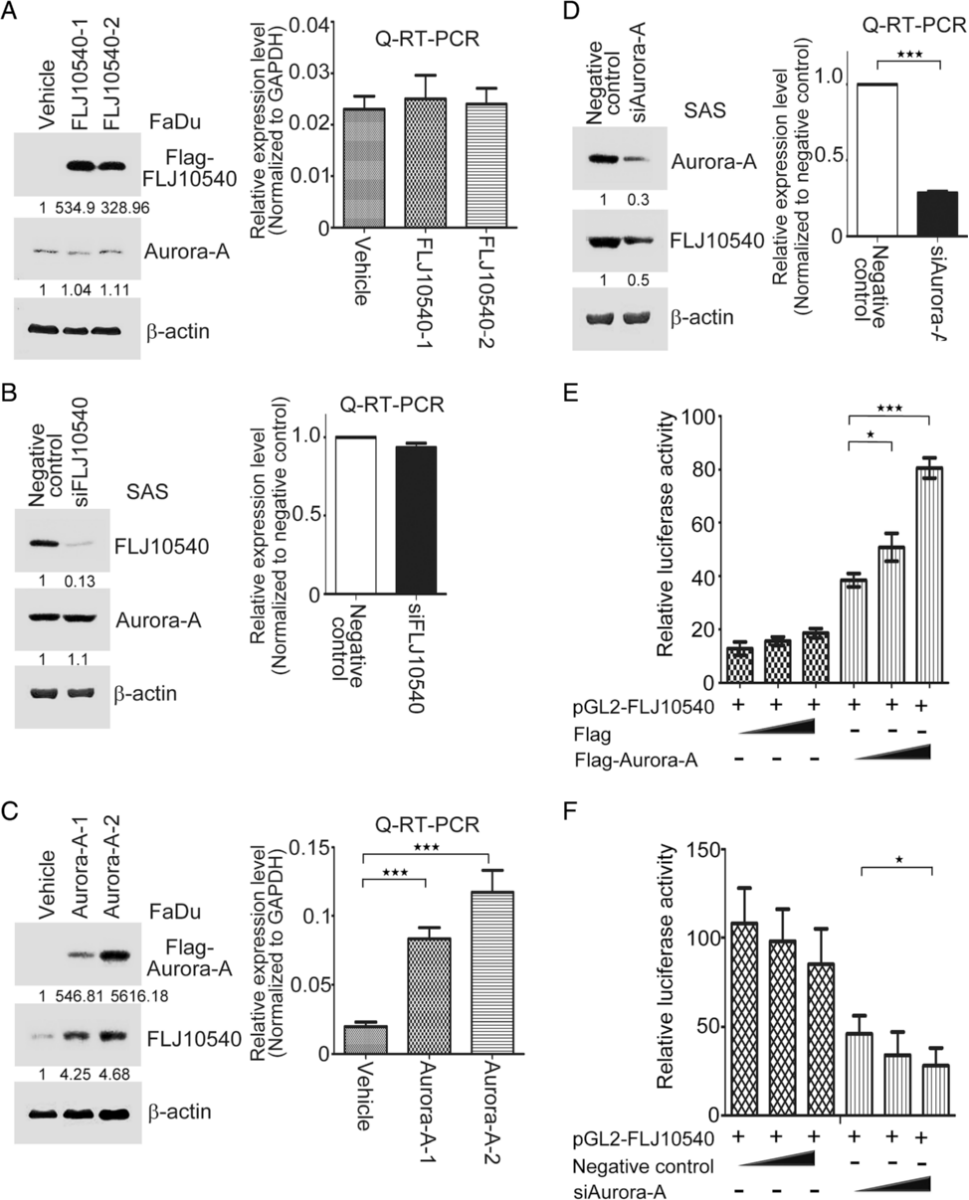
Aurora-A upregulates FLJ10540 in HNC. (**A** and **B**) Flag-tagged FLJ10540 stable clone of FaDu cells and siFLJ10540 transfectants of SAS cells were established. The cell lysates were subjected to immunoblot analysis with anti-Flag and FLJ10540 antibodies. β-actin is as an internal control. Aurora-A mRNA and protein expression levels in FLJ10540-overexpressing and vehicle cells were recognized by Western blotting and Q-RT-PCR. (**C** and **D**) Flag-tagged Aurora-A stable clone of FaDu cells and siAurora-A transfectants of SAS cells were established. The endogenous mRNA and protein of FLJ10540 were detected by Western blotting and Q-RT-PCR. (**E** and **F**) The luciferase assays were performed to detect promoter activities of FLJ10540 in cotransfected with in a dose-dependent manner of Flag, Flag-Aurora-A, negative control or siAurora-A. The FLJ10540 luciferase activity was normalized to Renilla activity. Data are representative of three independent experiments done in triplicates. Images were quantified with ImageJ software. Statistical analysis: *p < 0.05, ***p < 0.001.

To further explore the correlation between Aurora-A and FLJ10540 in HNC cells, we first inspected whether Aurora-A expression could be modulated by FLJ10540. FaDu cells were chosen for ectopic expressions of Aurora-A and FLJ10540 because of the low endogenous Aurora-A and FLJ10540 levels, and SAS cells were selected to generate the Aurora-A- and FLJ10540-knockdown systems owing to the higher endogenous Aurora-A and FLJ10540 expression levels. The data showed that both endogenous mRNA and protein expressions of Aurora-A were not altered in SAS and FaDu cells with FLJ10540-gain or -loss of functions, compared to the vehicle or negative control (Figure 2A and B) as detected by Q-RT-PCR and Western blotting. Next, we established overexpression and depletion of Aurora-A-HNC cell lines to determine the effect of FLJ10540 expression. As shown in the Figure 2C, compared with the vehicle control, the mRNA and protein expression levels of FLJ10540 were dramatically increased in overexpressed-Aurora-A-FaDu cells. Conversely, Aurora-A depletion led to reduction in FLJ10540 expression both on mRNA and protein levels in SAS cells (Figure 2D). To investigate whether FLJ10540 was transcriptionally induced by Aurora-A, the human promoter sequences of FLJ10540 fused luciferase reporter-based plasmid was generated and assessed the promoter activity of FLJ10540 in FaDu cells by Aurora-A modulation. Expressing Flag-Aurora-A or Flag alone was co-transfected with reporter plasmids respectively and luciferase activity determined after normalizing for transfection efficiency. Remarkably, Flag-Aurora-A overexpressing had 2- to 4-fold increases in FLJ10540 promoter activity compared with vehicle alone (Figure 2E). In contrast, the promoter activity of FLJ10540 was decreased while endogenous Aurora-A was knock-downed in SAS cells (Figure 2F). To confirm the binding of Aurora-A to the FLJ10540 promoter, chromatin-immunoprecipitation assay was performed. The results indicated that using α-Flag antibodies were able to specifically immunoprecipitate FLJ10540 promoter in cells transfected with Flag-Aurora-A; however, the control IgG isotype did not exhibit specific immunoprecipitation. Conversely, the FLJ10540 promoter region was not able to be precipitated in SAS cells transfected with Aurora-A siRNA (Additional file 1: Figure S1). Taken together, the earlier findings illustrate that in HNC cells, FLJ10540 is one of the downstream targets of Aurora-A.

### MLN8237 inhibits the expression of FLJ10540 and disrupts the FLJ10540-PI3K association in HNC cells

**Figure 3:**
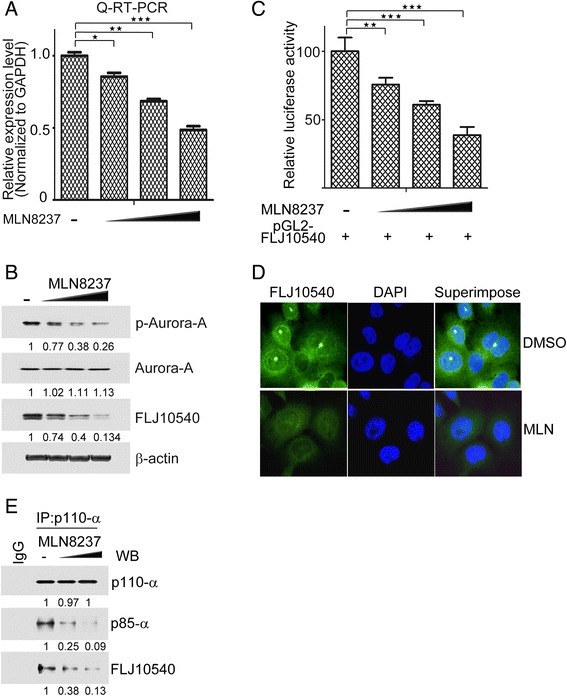
FLJ10540 expression and PI3K-FLJ10540 complex are restrained upon MLN8237 stimulation in HNC cells. (**A** and **B**) The mRNA and protein expression levels of FLJ10540 were examined by Q-RT-PCR and Western blotting in SAS cells in MLN8237 dose-dependent manner. The results were normalized against the expression level of *GAPDH* mRNA in each MLN8237-treated cell. Using the same panel, the total proteins were probed with antibodies against phosphorylated-Aurora-A, Aurora-A, FLJ10540 and β-actin. β-actin was used as a control. Data are representative of three independent experiments done in triplicate. (**C**) Luciferase assays were done to detect promoter activity of FLJ10540 in transfected FaDu cells in the presence or absence of MLN8237. (**D**) Unsynchronized HNC cells were fixed, and immunostained with antibodies against endogenous FLJ10540 or DAPI. Representative data are shown. (**E**) Co-immunoprecipitation of FLJ10540 and PI3K. Cells treated with or without MLN8237 and antibody of p110-α used for immunoprecipitation and representative western blot detection of p85-α and FLJ10540 were shown. Statistical analysis: *p < 0.05, **p < 0.01, ***p < 0.001.

MLN8237, an ATP-competitive and reversible inhibitor has been shown to inhibit Aurora-A activity in advanced malignancies [7,26]. To further confirm the positive correlation between Aurora-A activity and FLJ10540, we examined FLJ10540 expression by Q-RT-PCR and Western blotting under the condition of Aurora-A inhibition by MLN8237 treatment in SAS cell lines. As shown in Figure 3A and B, MLN8237 significantly reduced FLJ10540 mRNA and protein expressions in a concentration-dependent manner. To determine whether Aurora-A activity induction of FLJ10540 is regulated at the level of transcription, FLJ10540 reporter-based plasmids were transfected into FaDu cells under treatment of MLN8237 in a dose-dependent manner. As expected, the luciferase activity of FLJ10540 was suppressed upon MLN8237 treatment in a dose-dependent manner (Figure 3C). Next, we further investigated the protein expression of FLJ10540 in unsynchronized SAS cells by indirect immunofluorescence. It was indicated that indeed, endogenous FLJ10540 protein expression was decreased in HNC cells treated with MLN8237 (Figure 3D). Our previous data demonstrated that FLJ10540 may act as a scaffold protein to stabilize the PI3K complex in human cancer cells [16,18,24]. According to data described above, we inspired that FLJ10540 associated with PI3K is required Aurora-A activity. The data illustrated that the associated strength of PI3K-FLJ10540 complex was depended on Aurora-A activity (Figure 3E). These data suggest that the Aurora-A activity is critical for FLJ10540 expression and PI3K-FLJ10540 interaction in HNC cells.

### FLJ10540 mediates the function of Aurora-A on maintenance of malignant state *in vitro* and *in vivo*

**Figure 4:**
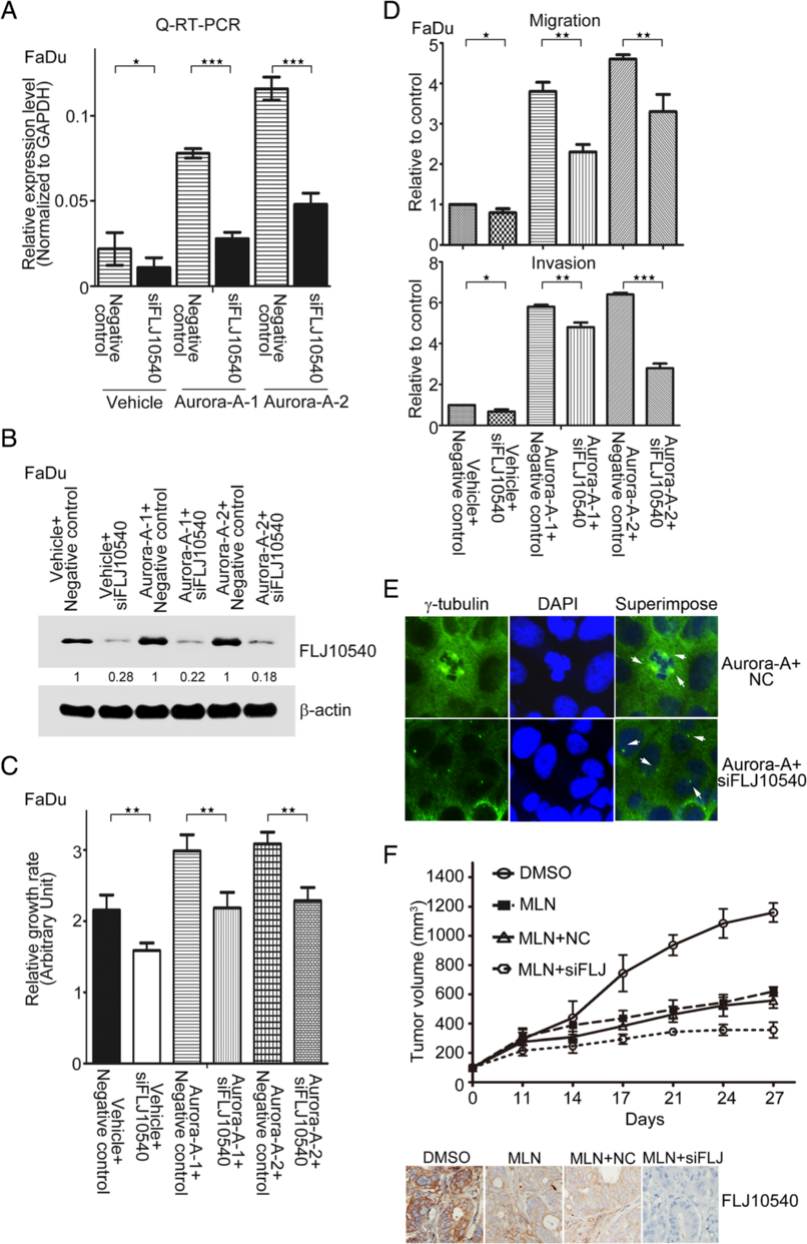
Depleted FLJ10540 attenuates Aurora-A elicited malignant phenotype changes *in vitro* and *in vivo*. (**A** and **B**) The mRNA and protein expression levels of FLJ10540 were determined by Q-RT-PCR and Western blotting in vehicle or overexpressing Aurora-A transfectants. The cell lysates of FaDu/vehicle-negative control, FaDu/vehicle-siFLJ10540, FaDu/Aurora-A-negative control, or FaDu/Aurora-A-siFLJ10540 transfectants were subjected to immunoblot analysis with anti-FLJ10540, and β-actin antibodies. Data are representative of three independent experiments done in triplicates. (**C** and **D**) Using the same panels, the proliferation, migration, and invasion assays were also performed by MTT assay and Transwell chambers. (**E**) Centrosome numbers were determined in Aurora-A stable cells transfected with negative control or siFLJ10540 in FaDu cells by using gamma-tubulin antibody. (**F**) Antitumor activity of MLN8237 plus siFLJ10540 in a SAS xenograft model. Nude mice bearing subcutaneously established SAS alone (n = 6), SAS/negative control (n = 6) or SAS/siFLJ10540 (n = 6) xenograft tumors and received the indicated treatments. Tumor growth was monitored and was shown as mean volumes ± SD. The paraffin-embedded tumor tissues were subjected to immunostaining for FLJ10540. Statistical analysis: *p < 0.05, **p < 0.01, ***p < 0.001.

To elucidate the role of FLJ10540 in Aurora-A-driven carcinogenesis on behavior change of HNC cells, the cell proliferation, migration and invasive assays were performed. First, Aurora-A stable cells transfected with negative control or siFLJ10540 were established. The mRNA and protein expression levels of FLJ10540 were dramatic reduced while endogenous FLJ10540 was depleted by FLJ10540-mediated siRNAs in steady expressed Aurora-A-FaDu cells (Figure 4A and B). Using the same panel, we observed that the abilities of cell growth, migration and invasion were inhibited in FaDu-Aurora-A stable cells transfected with FLJ10540 siRNA (Figure 4C and D). In addition, steady expressed Aurora-A/siFLJ10540 stable cells exhibited anti-tumor growth and metastasis *in vivo* (Additional file 2: Figure S2). Previous study reports that Aurora-A participates in centrosome amplification. To determine the role of FLJ10540 in this process, we analyzed centrosome amplification by immunofluorescence (gamma-tubulin) in overexpressed Aurora-A cells with FLJ10540 siRNA. The data indicated that nearly 10% of cells with abnormal centrosome amplification in overexpressed Aurora-A cells. However, the percentage of centrosome abnormalities dropped to less than 1% while conveyed to FLJ10540 depletion (Figure 4E, and Additional file 3: Figure S3). Next, to determine whether simultaneous inhibition of Aurora-A and FLJ10540 exhibited anti-tumor effect *in vivo*, xenograft models were used. SAS alone or transfected with negative control or siFLJ10540 cells were injected subcutaneously into the right flank of nude mice. Treatment with MLN8237/or DMSO was initiated when mean tumor volume was approximately 100 mm^3^ for all groups. As shown in Figure 4F, treatment with MLN8237 retarded tumor growth over the duration of the treatments, compared to DMSO group. Further, co-treatment with MLN8237 and siFLJ10540 resulted in significantly greater inhibition of tumor growth than treatment with MLN8237 alone. The mice tolerated all of the treatments without significant body weight difference was observed. In addition, the immunohistochemical staining showed that the expression of endogenous FLJ10540 was significantly decreased in treatment of MLN8237. Taken together, these results suggest that Aurora-A raises the development of HNC at least partly, through modulating FLJ10540 expression.

### Overexpression of FLJ10540 overturns the sensitivity of Aurora-A-depleted and Aurora-A-inactivated cells to cisplatin treatment

**Figure 5:**
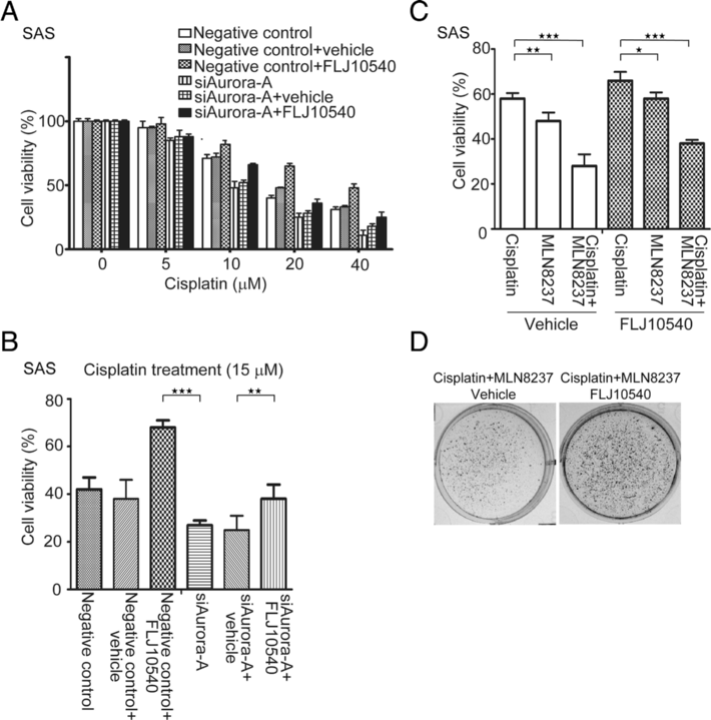
Reinforced FLJ10540 expression resists the cytotoxicity to cisplatin in depletion and inactivation cells of Aurora-A. (**A**) SAS/negative control or SAS/siAurora-A cells co-transfected with vehicle or FLJ10540 were incubated with increasing concentrations of cisplatin for 48 hour, and their percentage viability was measured and compared to that of untreated respective cells. (**B**) The cells were cultured in the presence of cisplatin (15 μM) for 48 hour, and their viability was measured. Vehicle and FLJ10540 transfectants were cultured in the presence or absence of cisplatin (15 μM) and/or MLN8237 for 48 hour (**C**) or 15 days (**D**) and total cell viability were assessed by MTT assay and soft agar. Statistical analysis: *p < 0.05, **p < 0.01, ***p < 0.001.

A recent studies have shown that Aurora-A contributes to the resistance to cisplatin in breast, pancreatic, esophageal squamous cell carcinoma, Acute myeloid leukemia, medulloblastoma, and ovarian cancer cells [7,27-29]. To gain insight the biological function of both Aurora-A and FLJ10540 on chemoresistance to cisplatin, we carried out MTT assay for assessing cell viability in siAurora-A transfected cells and siAurora-A/FLJ10540-overexpressed cells under cisplatin treatment. As shown in Figure 5A, the viability of siAurora-A cells was significantly more sensitive to cisplatin than negative control group in a concentration-dependent manner. Notably, in the presence of cisplatin, siAurora-A transfectants with FLJ10540 exhibited a lower sensitivity than siAurora-A transfectants or negative control to the cytotoxic effects of cisplatin (Figure 5A). In addition, the viability of siAurora-A cells was significantly lower than that of siAurora-A with FLJ10540-overexpressing cells after incubation with cisplatin (15 μM) for 48 hour (Figure 5B), indicating that increased FLJ10540 decreases the sensitivity of Aurora-A-depleted cells to cisplatin. Next, we used NLM8206 to investigate the effects of cisplatin in FLJ10540-overexpressing cells. As shown in Figure 5C, FLJ10540 transfectants incubated with MLN8206 (0.025nM) and cisplatin (15 μM) for 48 hour were significantly less viable than the same cells treated with cisplatin alone or MLN8206 alone. In addition, an assay of *in vitro* colony formation was used to confirm the result of MTT assay. As expected, FLJ10540 transfectants treated with MLN8237 and cisplatin in combination increased the colony-formation ability than that the vehicle control (Figure 5D). Together, these results demonstrate that the function of FLJ10540 modulated by Aurora-A expression or Aurora-A activity contributes to cisplatin resistance in HNC cells.

### Both MMP-7 and −10 expressions not only modulate by Aurora-A but also participate in Aurora-A-elicited cell motility in HNC cells

**Figure 6:**
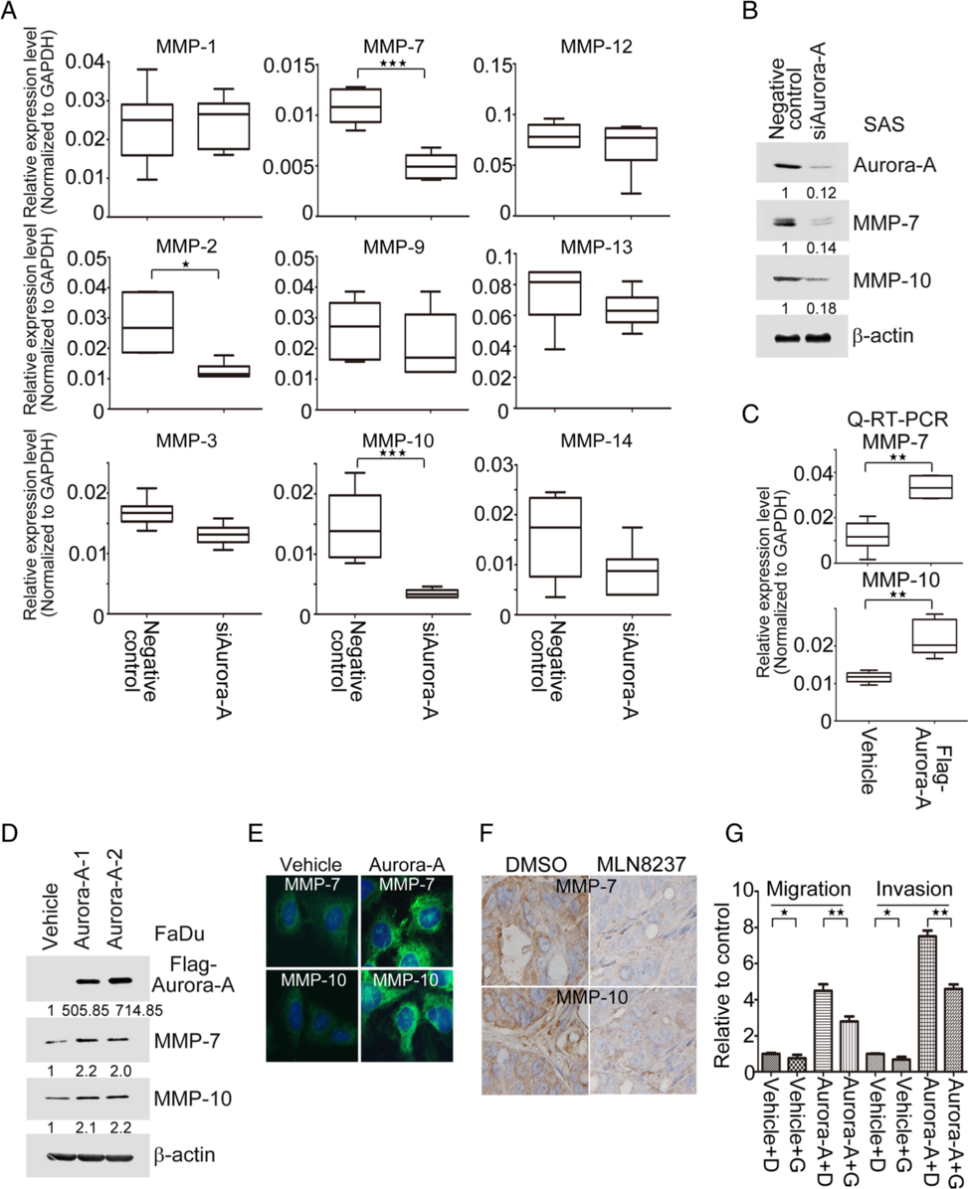
Aurora-A modulates MMP-7 and MMP-10 expression and secretion in HNC cells. The mRNA expression levels of *MMP-1, −2, −3, −7, −9, −10, −12, −13 and −14* (**A**) and protein expression profiles of MMP-7 and −10 (**B**) were examined by Q-RT-PCR and Western blotting in SAS/negative control and SAS/siAurora-A transfectants. (**C** and **D**) The endogenous mRNA and protein expression levels of *MMP-7,* and −*10* (**C**) were examined by Q-RT-PCR and Western blotting in FaDu/vehicle and FaDu/Aurora-A transfectants. (**E**) Unsynchronized Aurora-A transfectants were fixed, and immunostained with antibodies against endogenous MMP-7 and −10 or DAPI. Representative data are shown. (**F**) Using the Figure 4F xenograft model. The paraffin-embedded tumor tissues were subjected to immunostainings for MMP-7 and MMP-10. (**G**) The migration and invasion assays were performed by Transwell chambers in Aurora-A transfectants treated with GM6001 (3 μM). D: DMSO; G: GM6001. Statistical analysis: *p < 0.05, **p < 0.01, ***p < 0.001.

MMPs plays a crucial role in tumor invasion and metastasis for decades [30], and many MMPs contribute to HNC progression [31,32]. According to recent report, MMP-1, −2, −3, −7, −9, −10, −12, −13 and −14 are highly expressed in HNC and involved in HNC progression through invadopodia formation [30]. Here, we assessed these MMPs mRNA expression levels in Aurora-A depleted HNC cells. The results illustrated that only *MMP-7* and −*10* were dramatic suppressed in Aurora-A knockdown cells by Q-RT-PCR approach, compared to negative control (*p* < 0.05) (Figure 6A). Our Western blotting results are consistent with the observed mRNA expressions of *MMP-7* and −*10* in SAS cells transfected with siRNA of Aurora-A (Figure 6B). In contrast, ectopic expression of Aurora-A in FaDu cells was not only significantly enhanced the endogenous mRNA and protein expressions of MMP-7 and −10 (Figure 6C, D and E), but also increased the matricellular-MMP-7 and −10 levels in culture media by ELISA assay (Additional file 4: Figure S4). In xenograft tumor model, the MMP-7 and MMP-10 protein expressions were diminished in MLN8237 treated group compared to DMSO by immunohistochemical staining (Figure 6F). To further illustrate whether MMP-7 and −10 involves in Aurora-A-elicited cell motility, we used an MMP inhibitor, GM6001 to inhibit MMP-7 and MMP-10 expressions [33]. The data showed that the mRNA expression profiles of MMP-7 and −10 were decreased in Aurora-A transfectants under GM6001 treatment in a dose-dependent manner (Additional file 5: Figure S5). Additionally, using the same panel, Aurora-A-raised the abilities of cell migration and invasion were also dramatic suppressed (Figure 6G). Taken together, Aurora-A gives rise to the cell migration and invasion rely on both MMP-7 and −10 expressions in HNC cells.

### Aurora-A-mediated transcriptions and functions of both MMP-7 and −10 are dependent on FLJ10540 regulation

**Figure 7:**
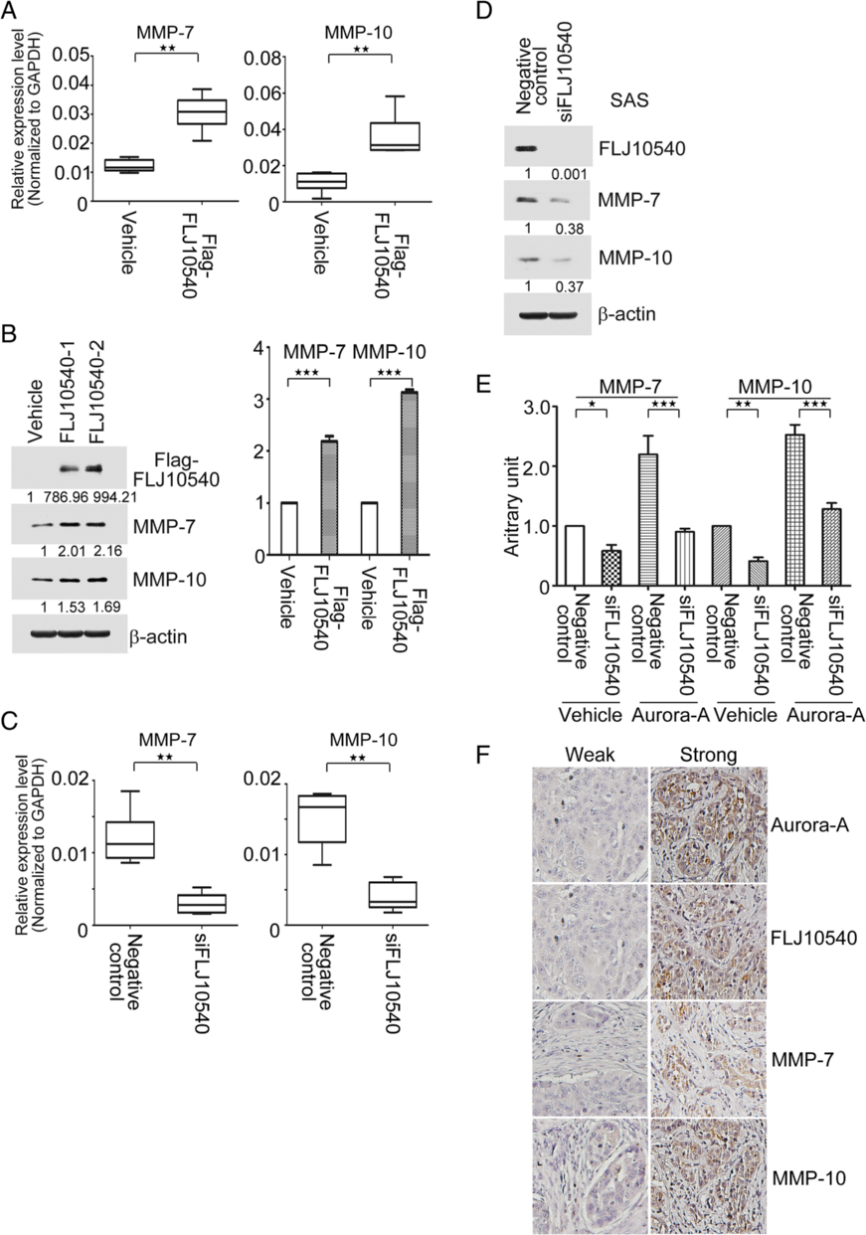
FLJ10540 not only promotes MMP-7 and −10 expressions through enforced Aurora-A expression, but also has a highly correlation with Aurora-A, MMP-7 and −10 expressions in human HNC tissues. FLJ10540 induced an increase in MMP-7, and −10 mRNA (**A**) and protein (**B**, left panel) levels in FaDu/FLJ10540 transfectants. (**B**, right panel) Conditioned media were prepared by incubating FaDu/vehicle and FaDu/FLJ10540 transfectants in serum-free media for 24 hour. MMP-7 and −10 in the samples were analyzed by ELISA. (**C** and **D**) The mRNA and protein expression profiles of MMP-7 and −10 were examined by Q-RT-PCR and Western blotting in SAS/negative control and SAS/siFLJ10540 transfectants. (**E**) The MMP-7 and −10 expression levels in conditioned media of vehicle and Aurora-A cotransfected with negative control or siFLJ10540 were assay by ELISA. (**F**) The FLJ10540, Aurora-A, MMP-7 and MMP-10 expressions in human HNC specimens were investigated by immunohistochemical staining. Photographs of weak and strong staining for FLJ10540, Aurora-A, MMP-7 and MMP-10 in the sections are shown. Statistical analysis: *p < 0.05, **p < 0.01, ***p < 0.001.

To test whether FLJ10540 contributes to the regulation of MMP-7 and −10 expressions in HNC, we investigated the effects of FLJ10540 on MMP-7 and −10 levels. As expected, both MMP-7 and −10 mRNA were up-regulated in FLJ10540-overexpressing transfectants by Q-RT-PCR (Figure 7A). Furthermore, FLJ10540 was not only enhanced the endogenous protein expression levels of both MMP-7 and −10, but also increased MMP-7 and −10 secretion in culture medium by Western blotting and ELISA approaches (Figure 7B). On the contrary, mRNA and protein expression profiles of both MMP-7 and −10 were remarkably decreased while FLJ10540 was depleted in HNC cell lines (Figure 7C and D). Furthermore, FLJ10540-elicited the abilities of cell migration and invasion were also dramatic suppressed while cells treated with GM6001 (Additional file 6: Figure S6). To investigate whether MMP-7 and −10 expression induced by Aurora-A is required FLJ10540, the secretory proteins of both MMP-7 and −10 were determined in Aurora-A transfectants in the present or absence of FLJ10540 siRNA. The data indicated that inhibition of FLJ10540 blocked MMP-7 and −10 secretions in response to Aurora-A modulation (Figure 7E). Ultimately, the Aurora-A-raised cell motility, growth, and chemoresistance were also dramatic suppressed while Aurora-A stable cells transfected with siFLJ10540 following GM6001 treatment, compared to Aurora-A/negative control/GM6001 (Additional file 7: Figure S7). Collectively, FLJ10540 is crucial for Aurora-A-induced MMP-7 and −10 transcriptions and functions in HNC cells.

### Aurora-A overexpression is correlated with FLJ10540, MMP-7 and MMP-10 expression in HNC tissue microarray

To determine the clinical significances of Aurora-A, FLJ10540, MMP-7, and −10 in HNC patients, we further examined their expressions in HNC specimens by immunohistochemistry in each tissue microarray including 70 cases of HNC samples and 10 cases of normal tissues. The relative expression levels of these markers were scored by two independent pathologists. In normal tissues, all of these molecules were expressed weak but detectable. As shown in Figure 7F, Aurora-A expression was correlated with FLJ10540, MMP-7 and MMP-10 expressions in HNC tissues. The correlation between each paired IHC scores of Aurora-A, FLJ10540, MMP-7, and MMP-10 were analyzed by Spearman’s rank tests. The result showed that there were positive correlations between Aurora-A and FLJ10540 (rho =0.776, p < 0.001), Aurora-A and MMP-7 (rho = 0.825, p < 0.001), Aurora-A and MMP-10 (rho = 0.728, p < 0.001), FLJ10540 and MMP-7 (rho = 0.795, p < 0.001), FLJ10540 and MMP-10 (rho = 0.768, p < 0.001) (Additional file 8 Table S1). Taken together, these results suggest that there is a significant positively correlation among Aurora-A, FLJ10540, MMP-7 and MMP-10 in human HNC.

## Discussion

The surgery, radiotherapy and chemotherapy used alone or in combination, are commonly employed for the treatment of HNC patients. Recently, molecular targeted therapies are in development with the goal of selective approaches to prevent the growth of HNC cells. Aurora-A and FLJ10540 overexpression has been explored in a variety of human cancers. However, before this study, the relationship between these two molecules in HNC has not been investigated.

In this study, we first demonstrated that both Aurora-A and FLJ10540 were not only commonly co-amplified, but also had a similar expression pattern in HNC tumor tissues and cell lines by using public accessory microarray database, Oncomine database, Q-RT-PCR, Western blotting and immunohistochemistry approaches. Gain and loss of function assays on Aurora-A indicated that Aurora-A regulates FLJ10540 expression via binding to the FLJ10540 promoter in HNC cells. In addition, inhibition of Aurora-A kinase activity by MLN8237 was not only significant decrease tumor growth and the FLJ10540 expression *in vitro* and *in vivo*, but also prevented the formation of FLJ10540-PI3K complex in HNC cells. Aurora-A overexpression in HNC cells elicited the characteristics of aggressive malignancy, such as proliferation, migration, invasion and centrosome abnormality were dramatic decreased, while endogenous FLJ10540 was suppressed. However, FLJ10540 overexpression significantly reversed the sensitivity of Aurora-A-depleted cells to cisplatin. Next, increased MMP-7 and MMP-10 activities by Aurora-A or FLJ10540 were required for increasing cell motility. In addition, Aurora-A elicited cell motility was dramatic suppressed, while FLJ10540 and MMP-7/MMP-10 were inhibited simultaneously comparing to Aurora-A alone. Finally, the expression of Aurora-A in HNC tissue microarray was correlated with elevated FLJ10540, MMP-7, and MMP-10 expressions. Taken together, we demonstrate a new molecular mechanism that aberrant Aurora-A expression in HNC plays a crucial role as positive activator of MMP-7 and MMP-10 via the function of FLJ10540 to regulate HNC progression.

It is known that overexpressed Aurora-A in cancer cells lead to carcinogenesis in multiple types of human cancers. Previous study has shown that Aurora-A participates in PI3K pathway for cancer cell survival [34]. Our previous data indicated that FLJ10540 could form a complex with PI3K for promoting tumor growth in HCC [16]. Recently, we had demonstrated that Aurora-A was activated in advanced stage of squamous cell carcinoma of head and neck cancer [12]. In addition, FLJ10540 expression was correlated with aggressiveness of oral cavity squamous cell carcinoma [19]. Thus, these results suggest that Aurora-A and FLJ10540 may have functionally linked in head and neck cancer patients. In this study, we first demonstrated that Aurora-A regulated FLJ10540 expression in transcriptional and post-transcriptional levels in HNC cells, suggesting that Aurora-A might regulate PI3K activation through the FLJ10540 function. As expect, Aurora-A kinase activity inhibition in HNC cells led to significant decrease the FLJ10540-PI3K complex formation as well as FLJ10540 protein levels by using Aurora-A inhibitor of MLN8237. The decreased FLJ10540-PI3K association was also observed while Aurora-A was depleted by Aurora-A-mediated siRNA (data not shown). This is the first time to unravel how Aurora-A participates in PI3K signaling pathway in human cancer. These results not only demonstrated that Aurora-A was an upstream regulator of FLJ10540, but also revealed cross-talk between Aurora-A and FLJ10540 in modulating PI3K pathway.

It is noteworthy that down-regulation of Aurora-A or its targets improves chemosensitivity of human cancers [35]. However, whether FLJ10540 involved in cisplatin chemoresistance in Aurora-A depleted cells has not been reported. In the present study, compared with the corresponding control cells, the viability of Aurora-A depleted HNC cells was reduced under cisplatin treatment in a dose-dependent. Moreover, FLJ10540 overexpressed in Aurora-A-depleted HNC cells or FLJ10540 transfectants treated with MLN8237 could overcome the chemosensitive characteristic to cisplatin compared to Aurora-A-depleted cells or vehicle control. Thus, these results suggest that overexpressed Aurora-A-raised chemoresistant to cisplatin are at least partly, through regulating FLJ10540 expression.

According to current concept, MMP expression could maintain tumor microenvironment such as stroma cells, so inhibited MMP expression would be more effective in a cancer treatment [33,36]. Several studies reported that Aurora-A enhances cancer cell metastasis through MMP-2 expression in human cancers [37,38]. By using Q-RT-PCR screening approach, we identified that MMP-7 and MMP-10 expressions, but not MMP-2 were dramatic increased by both Aurora-A and FLJ10540 regulations in HNC cells. In addition, MMP-7- and MMP-10-elicited HNC cell motility were regulated by Aurora-A-FLJ10540 expression. This is the first study to unravel that one of the important upstream regulators of FLJ10540 and MMP-7/MMP-10 may be regulated by Aurora-A in HNC cells. Finally, the immunohistochemical analysis showed a significant correlation that overexpression of Aurora-A was not only elevated expression of FLJ10540, but also increased MMP-7 and MMP-10 expression in HNC tissues. However, the molecule mechanisms of FLJ10540 regulated MMP-7 and MMP-10 expression via Aurora-A modulation need to be further elucidated.

In summary, this study highlights the importance of Aurora-A signaling pathway in the biology of HNC at clinical tissue, cell line and murine tissue levels. The impact of suppression of Aurora-A pathway is imposed across all translational related characteristics of HNC cells such as viability, invasiveness, chemoresistance and *in vivo* tumorigenesis.

## Conclusions

**Figure 8:**
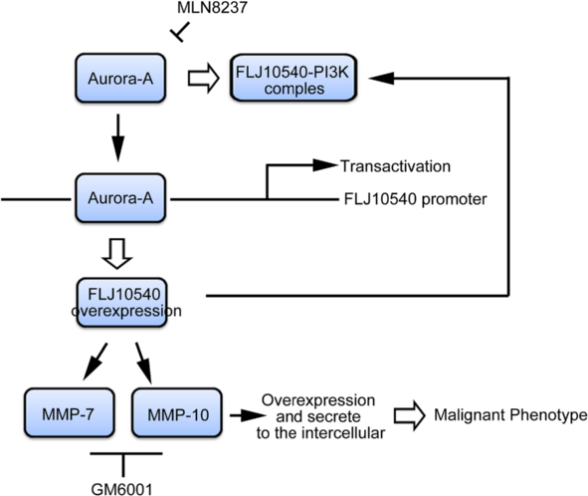
Schematic presentation for the molecular mechanism by which MMP-7 and MMP-10 expressions is regulated by the Aurora-A/FLJ10540 pathway, resulting in the proliferation, migration, invasion, and chemoresistance of HNC cells.

This study for the first time provides a new insight into a novel relationship between Aurora-A and FLJ10540 expressions on MMP-7 and MMP-10 activation. This Aurora-A-FLJ10540-MMP-7/MMP-10 axis is involved in cancer cell proliferation, migration, invasion and chemoresistance (Figure 8). Therefore, targeting Aurora-A-FLJ10540-MMP-7/MMP-10 axis with Aurora-A inhibitor could be an effective therapeutic approach in HNC.

## Additional files

Additional file 1: Figure S1. Aurora-A binds to the promoter region of FLJ10540 in HNC cells. Chromatin immunoprecipitation assay was performed using Flag or control antibodies (A) or Aurora-A antibody (B) to pull down DNA fragment on the FLJ10540 promoter from FaDu and SAS transfected with siAurora-A cells. The FLJ10540 promoter element was detected by Q-RT-PCR. Statistical analysis: ***p < 0.001. Additional file 2: Figure S2. Suppression of endogenous FLJ10540 postpones Aurora-A elicited tumor growth and metastasis *in vivo*. (A) Tumor growth evaluation of FaDu-vehicle and FaDu-Aurora-A cells transfected with negative control or siFLJ10540 in xenograft model. Nude mice bearing subcutaneously established FaDu-vehicle-negative control (n = 6), FaDu-vehicle-siFLJ10540 (n = 6), FaDu-Aurora-A-negative control (n = 6), and FaDu-Aurora-A-siFLJ10540 (n = 6) xenograft tumors. Tumor growth was monitored and was shown as mean volumes ± SD. (B) Using the same panel, lung metastasis assay and histological analysis from nude mice. T, metastatic nodule. Additional file 3: Figure S3. The quantification of gamma-tubulin in overexpressing Aurora-A stable cells transfected with negative control or FLJ10540 siRNA. Ratio of gamma-tubulin localized at centrosome was quantified from 20 images per condition. Statistical analysis: ***p < 0.001. Additional file 4: Figure S4. The matricellular proteins of MMP-7 and MMP-10 are increased in Aurora-A stable transfectants. Conditioned media were prepared by incubating FaDu/vehicle and FaDu/Aurora-A transfectants, in serum-free media for 24 h. The secrete proteins of MMP-7 and −10 were analyzed by ELISA. Statistical analysis: ***p < 0.001. Additional file 5: Figure S5. GM6001 inhibits *MMP-7* and −*10* mRNA expressions in Aurora-A stable cells in HNC. The mRNA expression levels of *MMP-7* and −*10* were examined by Q-RT-PCR in FaDu/vehicle, and FaDu/Aurora-A transfectants treated with DMSO or GM6001 in a dose-dependent manner. Statistical analysis: **p < 0.01, ***p < 0.001. Additional file 6: Figure S6. GM6001 inhibits FLJ10540-elicited cell motility in HNC. The migration and invasion assays were performed by Transwell chambers in FLJ10540 transfectants treated with GM6001 (3 μM). Statistical analysis: ***p < 0.001. Additional file 7: Figure S7. GM6001 and siFLJ10540 were synergistic inhibition the Aurora-A-elicited cell motility, growth, and chemoresistance in HNC. (A) The migration and invasion assays were performed by Transwell chambers in Aurora-A/FaDu and vehicle cells transfected and/or negative control or siFLJ10540 following treated with GM6001. (B) The colony formation was assay in Aurora-A/FaDu transfected with negative control or siFLJ10540 following treated with GM6001. (C) Using the same panel B, cells were incubated with cisplatin for 48 h, and their percentage viability was measured. Statistical analysis: *p < 0.05, **p < 0.01. Additional file 8: Table S1. The correlation between Aurora-A, FLJ10540, MMP-7 and MMP-10 expressions in HNC.
