# Supplementary figures and images for: Suppression of Aurora-A-FLJ10540 signaling axis prohibits the malignant state of head and neck cancer
Source: Mol Cancer. 2015 Apr 12;14:83. doi: 10.1186/s12943-015-0348-7 (PMC4403844; doi:10.1186/s12943-015-0348-7)

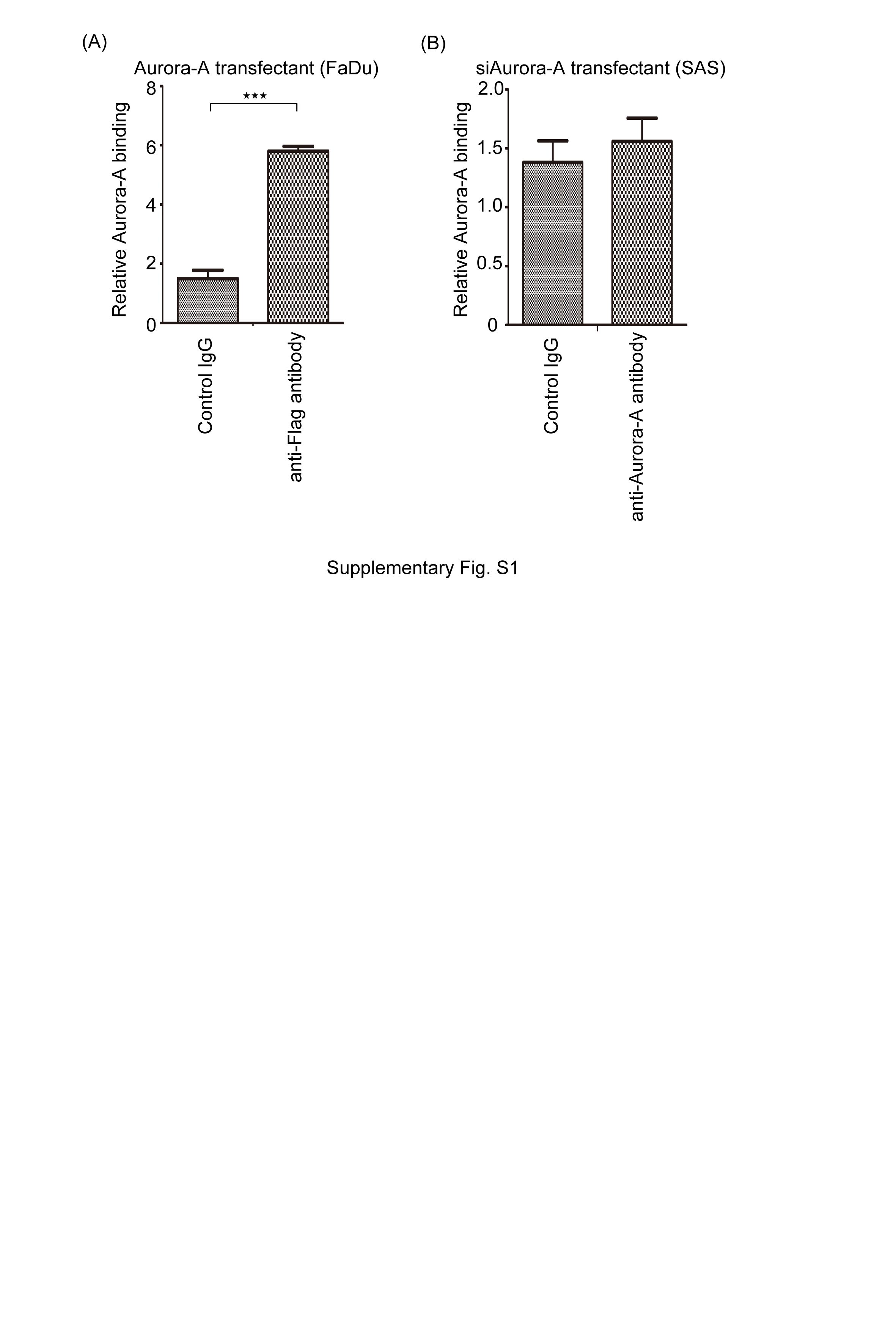

Supplement: Additional file 1: Figure S1. — Aurora-A binds to the promoter region of FLJ10540 in HNC cells. Chromatin immunoprecipitation assay was performed using Flag or control antibodies (A) or Aurora-A antibody (B) to pull down DNA fragment on the FLJ10540 promoter from FaDu and SAS transfected with siAurora-A cells. The FLJ10540 promoter element was detected by Q-RT-PCR. Statistical analysis: ***p < 0.001. [file 12943_2015_348_MOESM1_ESM.tiff]

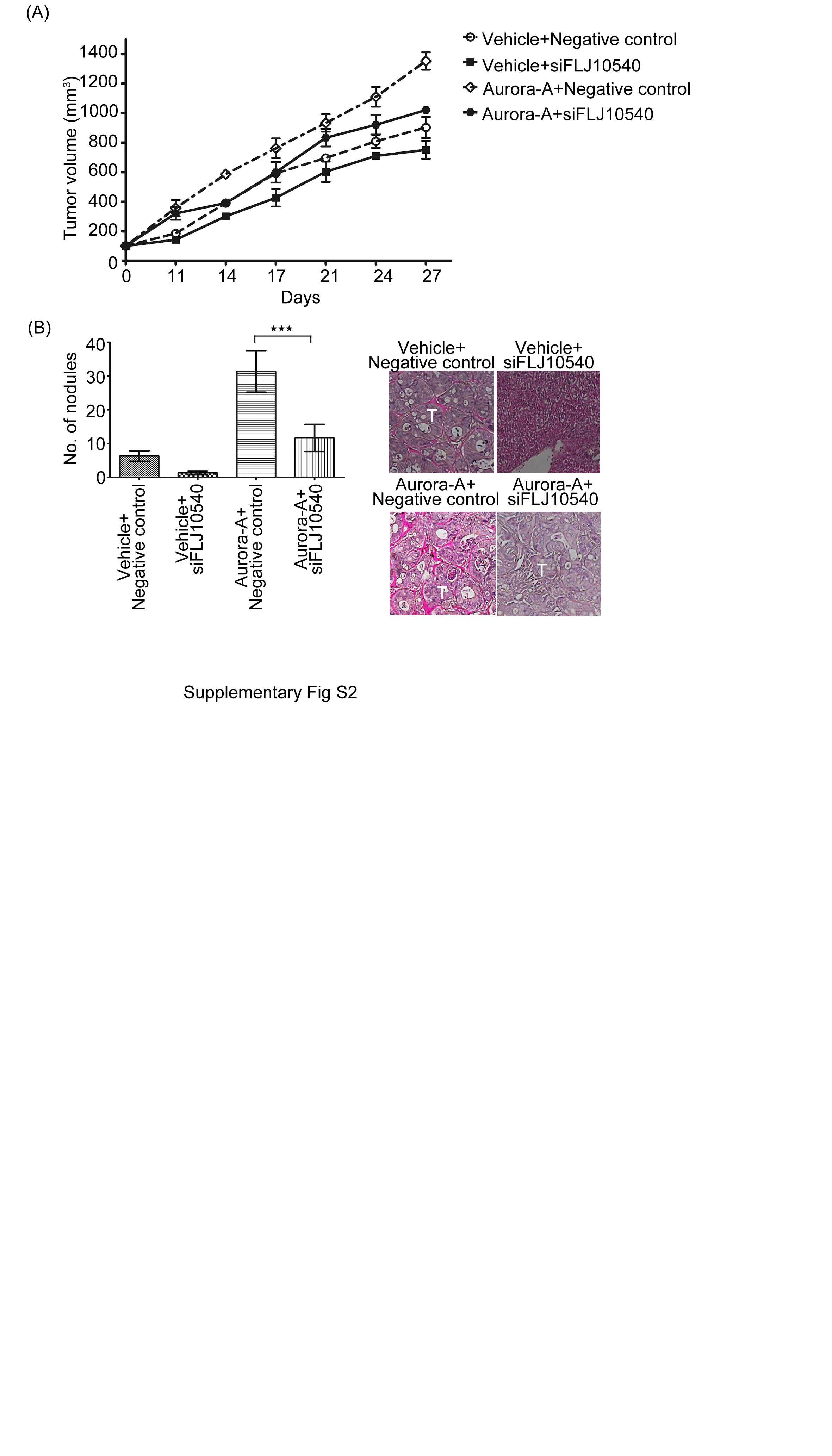

Supplement: Additional file 2: Figure S2. — Suppression of endogenous FLJ10540 postpones Aurora-A elicited tumor growth and metastasis in vivo. (A) Tumor growth evaluation of FaDu-vehicle and FaDu-Aurora-A cells transfected with negative control or siFLJ10540 in xenograft model. Nude mice bearing subcutaneously established FaDu-vehicle-negative control (n = 6), FaDu-vehicle-siFLJ10540 (n = 6), FaDu-Aurora-A-negative control (n = 6), and FaDu-Aurora-A-siFLJ10540 (n = 6) xenograft tumors. Tumor growth was monitored and was shown as mean volumes ± SD. (B) Using the same panel, lung metastasis assay and histological analysis from nude mice. T, metastatic nodule. [file 12943_2015_348_MOESM2_ESM.tiff]

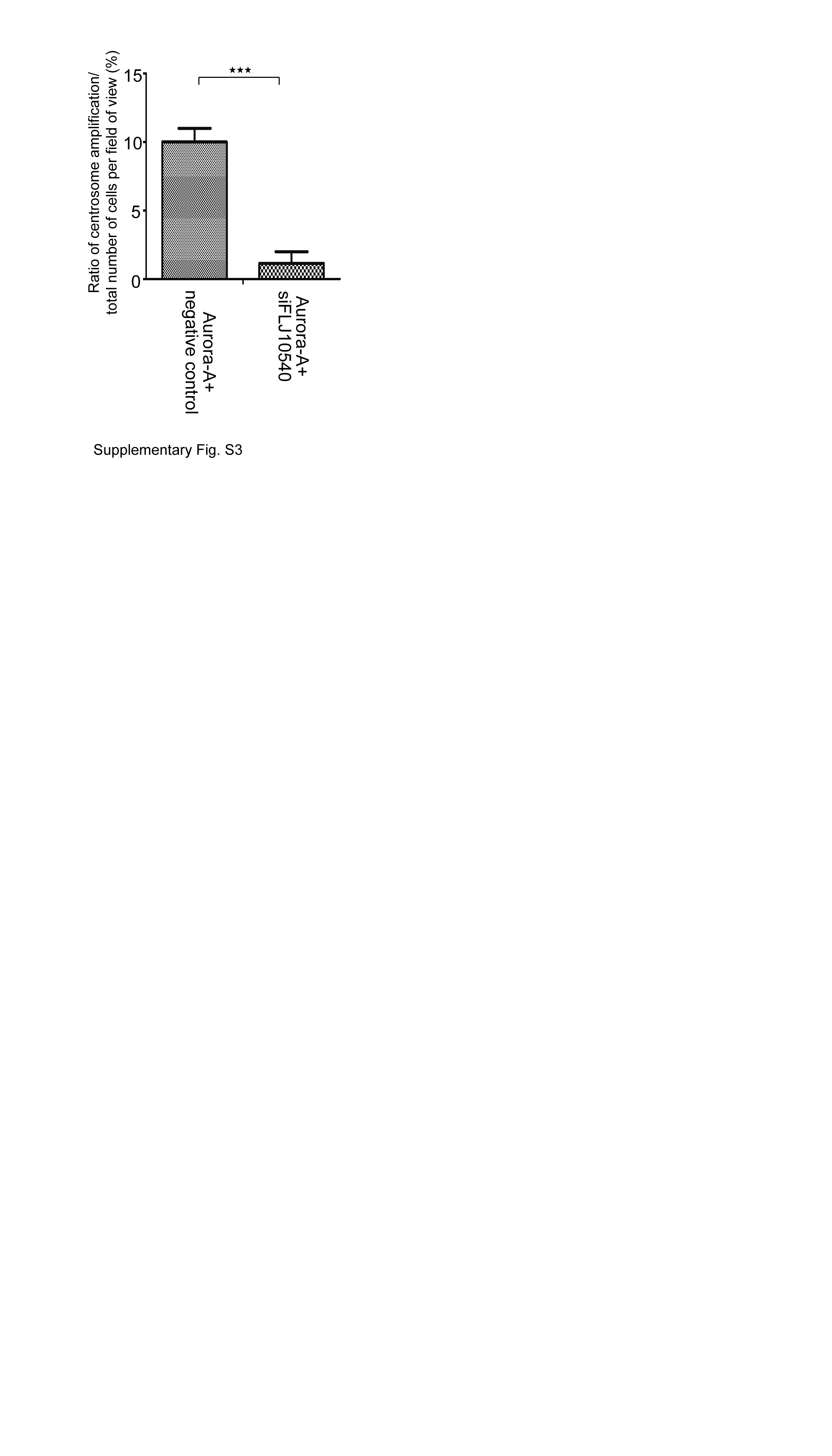

Supplement: Additional file 3: Figure S3. — The quantification of gamma-tubulin in overexpressing Aurora-A stable cells transfected with negative control or FLJ10540 siRNA. Ratio of gamma-tubulin localized at centrosome was quantified from 20 images per condition. Statistical analysis: ***p < 0.001. [file 12943_2015_348_MOESM3_ESM.tiff]

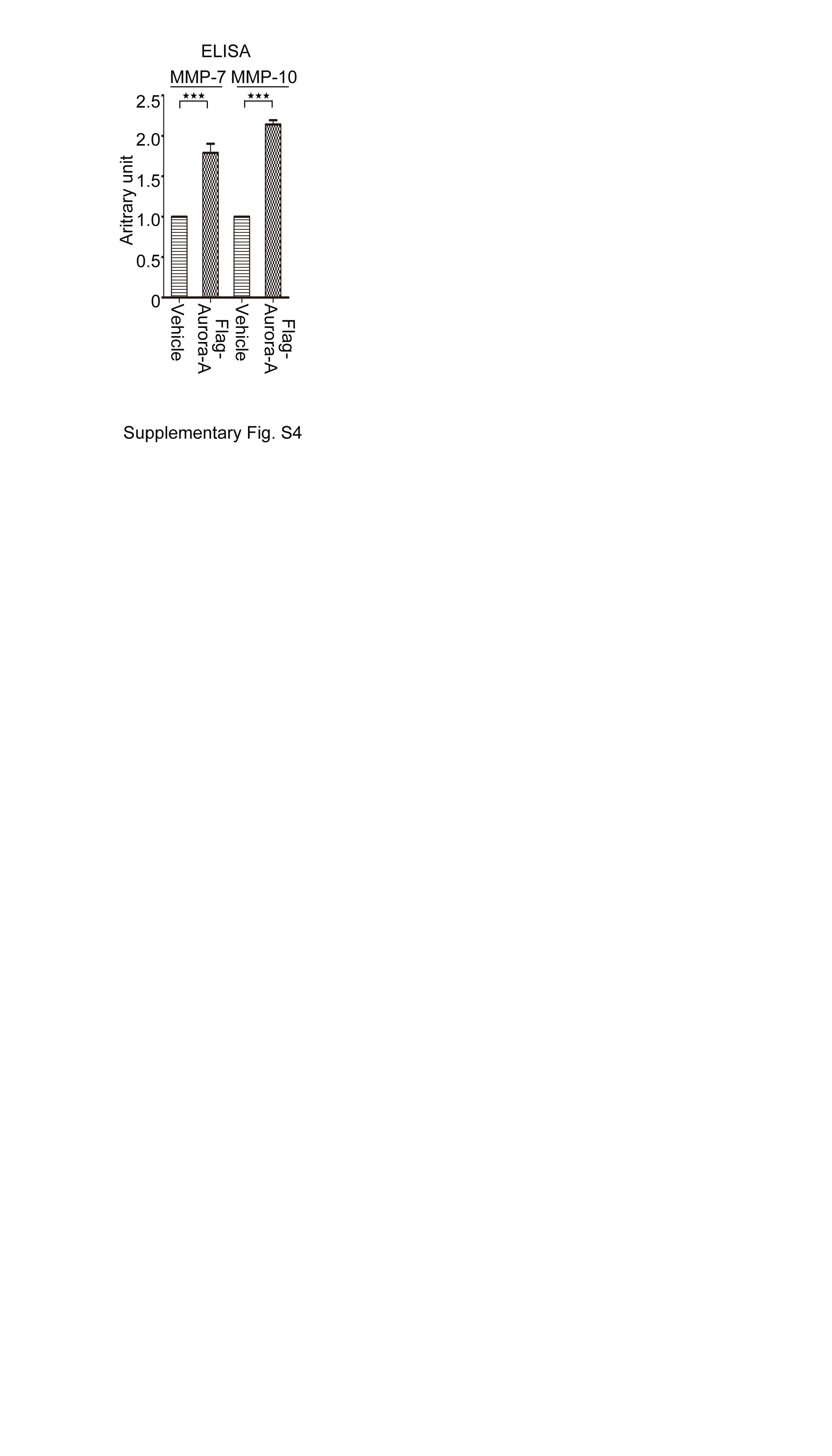

Supplement: Additional file 4: Figure S4. — The matricellular proteins of MMP-7 and MMP-10 are increased in Aurora-A stable transfectants. Conditioned media were prepared by incubating FaDu/vehicle and FaDu/Aurora-A transfectants, in serum-free media for 24 h. The secrete proteins of MMP-7 and −10 were analyzed by ELISA. Statistical analysis: ***p < 0.001. [file 12943_2015_348_MOESM4_ESM.tiff]

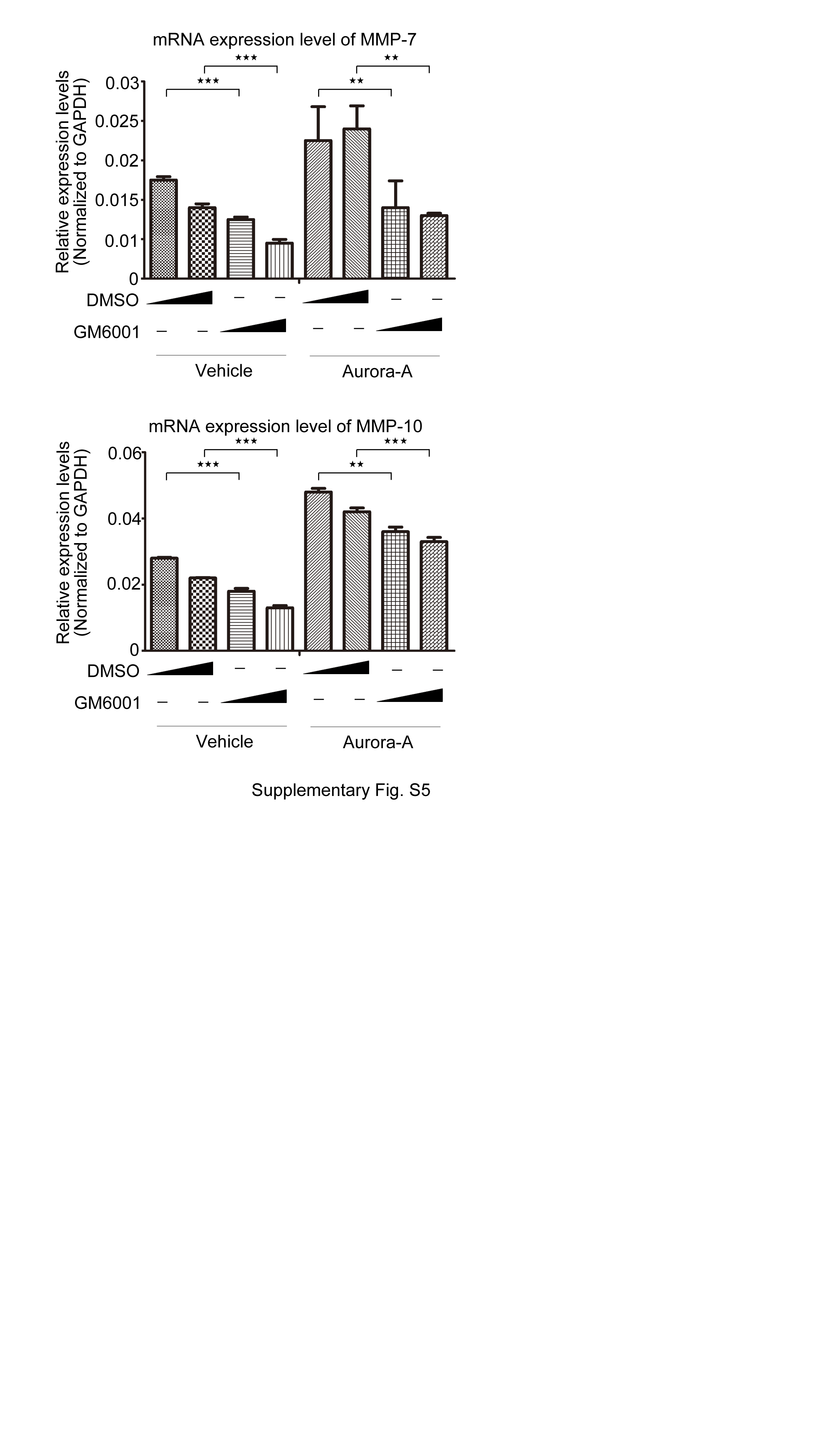

Supplement: Additional file 5: Figure S5. — GM6001 inhibits MMP-7 and −10 mRNA expressions in Aurora-A stable cells in HNC. The mRNA expression levels of MMP-7 and −10 were examined by Q-RT-PCR in FaDu/vehicle, and FaDu/Aurora-A transfectants treated with DMSO or GM6001 in a dose-dependent manner. Statistical analysis: **p < 0.01, ***p < 0.001. [file 12943_2015_348_MOESM5_ESM.tiff]

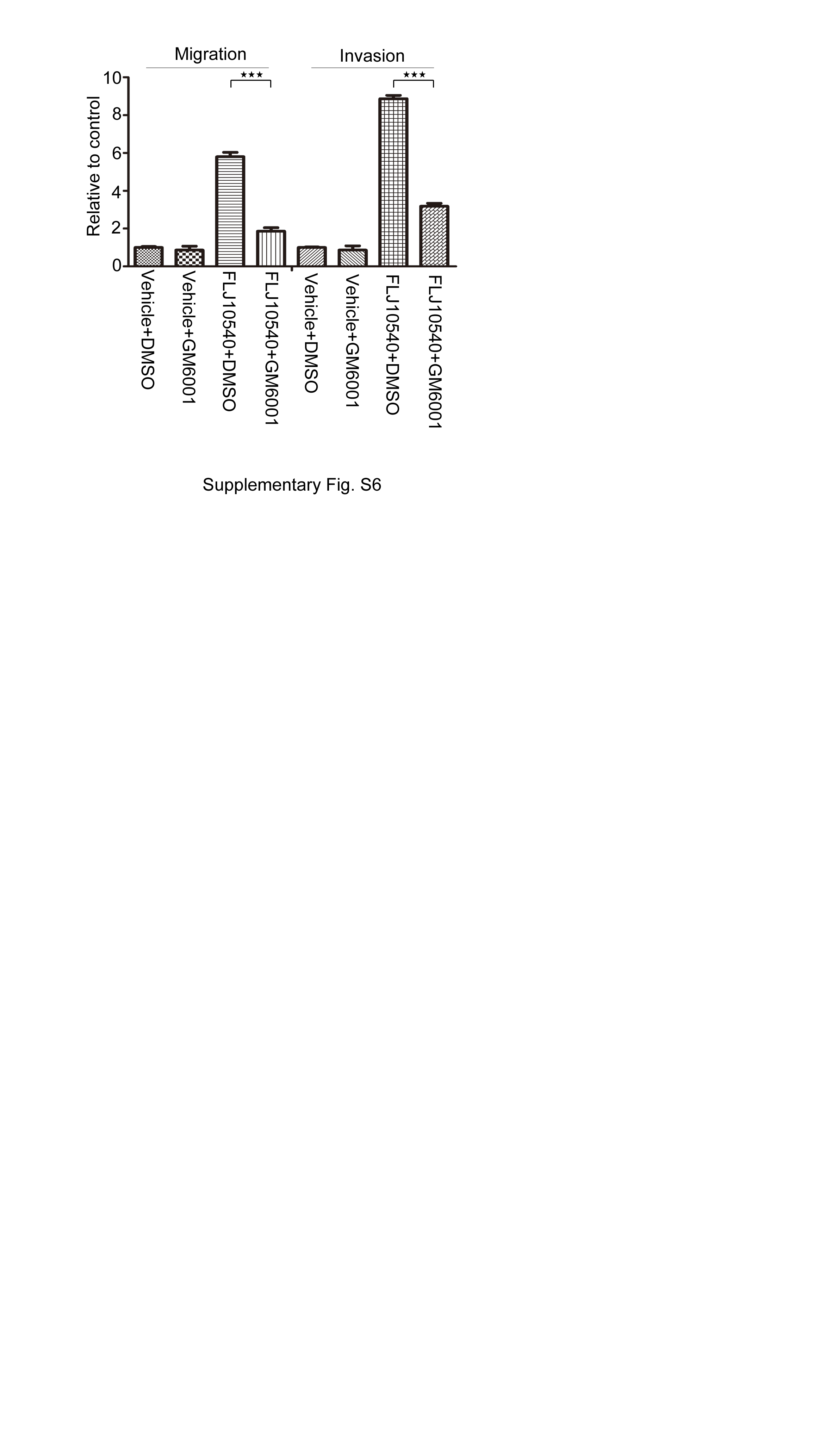

Supplement: Additional file 6: Figure S6. — GM6001 inhibits FLJ10540-elicited cell motility in HNC. The migration and invasion assays were performed by Transwell chambers in FLJ10540 transfectants treated with GM6001 (3 μM). Statistical analysis: ***p < 0.001. [file 12943_2015_348_MOESM6_ESM.tiff]

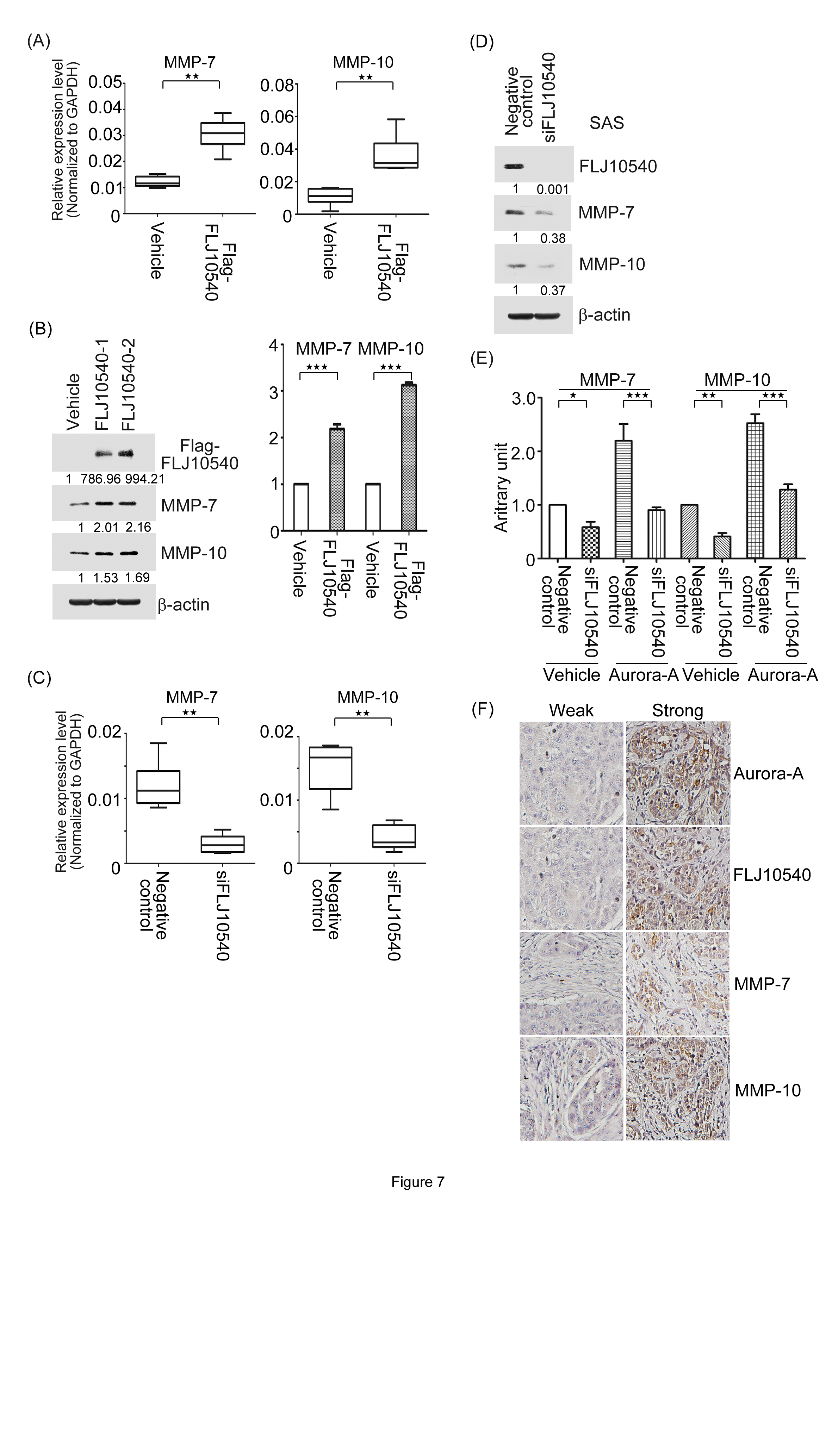

Supplement: Additional file 7: Figure S7. — GM6001 and siFLJ10540 were synergistic inhibition the Aurora-A-elicited cell motility, growth, and chemoresistance in HNC. (A) The migration and invasion assays were performed by Transwell chambers in Aurora-A/FaDu and vehicle cells transfected and/or negative control or siFLJ10540 following treated with GM6001. (B) The colony formation was assay in Aurora-A/FaDu transfected with negative control or siFLJ10540 following treated with GM6001. (C) Using the same panel B, cells were incubated with cisplatin for 48 h, and their percentage viability was measured. Statistical analysis: *p < 0.05, **p < 0.01. [file 12943_2015_348_MOESM7_ESM.tiff]
