# Supplementary material for: Suppression of Aurora-A-FLJ10540 signaling axis prohibits the malignant state of head and neck cancer
Source: Mol Cancer. 2015 Apr 12;14:83. doi: 10.1186/s12943-015-0348-7 (PMC4403844; doi:10.1186/s12943-015-0348-7)
Supplement: Additional file 8: Table S1. — The correlation between Aurora-A, FLJ10540, MMP-7 and MMP-10 expressions in HNC. [file 12943_2015_348_MOESM8_ESM.doc]

**Additional file 8 Table S1. The correlation between Aurora A, FLJ10540, MMP-7 and MMP-10 expressions in HNC**

|  |  | **Aurora-A** | **FLJ10540** | **MMP-7** | **MMP-10** |
| --- | --- | --- | --- | --- | --- |
| **Aurora-A** | Spearman’s rank correlation | 1 |  |  |  |
|  | Sig. (2-tailed) | . |  |  |  |
|  | Number | 70 |  |  |  |
| **FLJ10540** | Spearman’s rank correlation | 0.776 | 1 |  |  |
|  | Sig. (2-tailed) | <0.0001 | . |  |  |
|  | Number | 70 | 70 |  |  |
| **MMP-7** | Spearman’s rank correlation | 0.825 | 0.795 | 1 |  |
|  | Sig. (2-tailed) | <0.0001 | <0.0001 | . |  |
|  | Number | 70 | 70 | 70 |  |
| **MMP-10** | Spearman’s rank correlation | 0.728 | 0.768 | 0.0086 | 1 |
|  | Sig. (2-tailed) | <0.0001 | <0.0001 | 0.186 | . |
|  | Number | 70 | 70 | 70 | 70 |
